# Multi‐disciplinary, simulation‐based, standardised trauma team training within the Victorian State Trauma System

**DOI:** 10.1111/1742-6723.14068

**Published:** 2022-09-01

**Authors:** Mark C Fitzgerald, Michael Noonan, Emma Lim, Joseph K Mathew, Ellaine Boo, Helen E Stergiou, Yesul Kim, Stephanie Reilly, Christopher Groombridge, Amit Maini, Kim Williams, Biswadev Mitra

**Affiliations:** ^1^ National Trauma Research Institute The Alfred Hospital Melbourne Victoria Australia; ^2^ Trauma Service The Alfred Hospital Melbourne Victoria Australia; ^3^ Central Clinical School Monash University Melbourne Victoria Australia; ^4^ Emergency and Trauma Centre The Alfred Hospital Melbourne Victoria Australia; ^5^ School of Public Health and Preventive Medicine Monash University Melbourne Victoria Australia

**Keywords:** education, multi‐disciplinary communication, simulation training, trauma team, wounds and injuries

## Abstract

**Objective:**

Inconsistency in the structure and function of team‐based major trauma reception and resuscitation is common. A standardised trauma team training programme was initiated to improve quality and consistency among trauma teams across a large, mature trauma system. The aim of this manuscript is to outline the programme and report on the initial perception of participants.

**Methods:**

The Alfred Trauma Team Reception and Resuscitation Training (TTRRT) programme commenced in March 2019. Participants included critical care and surgical craft group members commonly involved in trauma teams. Training was site‐specific and included rural, urban and tertiary referral centres. The programme consisted of prescribed pre‐learning, didactic lectures, skill stations and simulated team‐based scenarios. Participant perceptions of the programme were collected before and after the programme for analysis.

**Results:**

The TTRRT was delivered to 252 participants and 120 responses were received. Significant improvement in participant‐reported confidence was identified across all key topic areas. There was also a significant increase in both confidence and clinical exposure to trauma team leadership roles after participation in the programme (from 53 [44.2%] to 74 [61.7%; *P* = 0.007]). This finding was independent of clinician experience.

**Conclusions:**

A team‐based trauma reception and resuscitation education programme, introduced in a large, mature trauma system led to positive participant‐reported outcomes in clinical confidence and real‐life team leadership participation. Wider implementation combined with longitudinal data collection will facilitate correlation with patient and staff‐centred outcomes.

## Graphical abstract

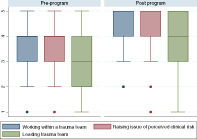

**Key findings**

- A team‐based trauma reception and resuscitation education programme, introduced into a large, mature trauma system led to positive participant‐reported outcomes.

## Introduction

The Victorian State Trauma System (VSTS) facilitates the management and treatment of major trauma patients in the state of Victoria, Australia. Following the introduction of the VSTS in 1999, trauma mortality and morbidity have been reduced significantly.^1^

Since the introduction of large‐scale trauma systems, hospital‐based trauma reception and resuscitation have been delivered by multi‐disciplinary teams consisting of various combinations of medical, nursing, paramedical and technical staff performing multiple procedures and evaluations simultaneously.^2^ In the hectic stages of trauma resuscitation, errors are common. There is evidence that the introduction of trauma teams can significantly improve patient outcomes.^3^, ^4^ However, even in mature, advanced trauma centres, error‐free resuscitations remain uncommon.^9^

Although teams make fewer mistakes than individuals,^4^ bringing individual ‘experts’ together to perform a specified task does not automatically ensure that they will function effectively as a team.^5^ Effective trauma teamwork depends on a willingness of clinicians from diverse backgrounds to effectively communicate and collaborate to achieve shared goals. In addition, effective trauma teams require individuals to be self‐reflective and be open to learn from shared experiences; collectively these are known as ‘non‐technical skills’. It follows that ineffective trauma team performance cannot be attributed solely to inadequate knowledge or skills of individual team members, but from deficits in the ‘non‐technical skills’ of the team.^6^, ^7^ ‘Training’ trauma teams to improve both technical and non‐technical skills have therefore been strongly supported by most jurisdictions with trauma systems.^7^

Simulation‐based training has been shown to be effective method of training for clinicians working in Operating Rooms, Intensive Care Units and EDs.^8^, ^9^, ^10^, ^11^ Trauma programmes focussing on single ‘craft groups’, such as the Advanced Trauma Life Support (ATLS©) course (medical focussed) of the American College of Surgeons and the Trauma Nursing Programme (nursing focussed)^12^ are also examples. However, training in multi‐disciplinary teams reflects real‐world scenarios and is likely to be of greater benefit.^7^, ^13^, ^14^, ^15^

Up to 30% of major trauma patients in Victoria are treated primarily in regional and urban hospitals prior to transfer to a major trauma centre. In 2018, it was recognised that there was no consistent approach across the State of Victoria for reception and resuscitation management within trauma teams. To address the gap and to achieve further improvements in patients' outcomes, a standardised formal trauma team training programme titled ‘Trauma Team Reception and Resuscitation Training (TTRRT)’ was developed by trauma staff at The Alfred Hospital, one of two Victorian adult major trauma centres.

The aims and objectives of the TTRRT programme were to improve the reception, resuscitation, and outcomes of trauma patients in Victoria by:

1. Implementing a standardised, contextualised, simulated multi‐specialty trauma team training course at one adult major trauma centre and five regional trauma centres for staff involved in trauma reception and resuscitation over a 2‐year period.
2. Identifying and establishing change champions throughout training and conduct ‘train the trainer programmes’.
3. Conducting qualitative assessments of participants using a validated, self‐reported and observational tool pre, immediately post and 12 weeks post training.
4. Evaluating the programme with a concurrent research study to evaluate quantitative and qualitative end­points of each facet of training.

The aim of this manuscript is to outline the intervention of TTRRT and report on the initial perceptions of participants.

## Methods

### Participants and settings

The TTRRT programme commenced on 7 March 2019. Participants were invited from key craft groups managing trauma reception and resuscitation. Those included were medical and nursing staff from emergency medicine, anaesthesia, intensive care, trauma and surgical specialty areas. Participant experience and primary location of work varied widely and included staff from tertiary referral, urban and rural settings. Participation was voluntary and without monetary cost to individuals. Funding for the programme was provided by the Transport Accident Commission.

There were 27 programmes delivered across one major trauma centre (*n* = 17), two large suburban hospitals (*n* = 3) and four regional hospitals (*n* = 7). Training occurred on‐site at participating hospitals to facilitate staff access, and local medical or nursing students volunteered to act as simulated patients. Where clinically possible, simulation training took place *in situ* in the resuscitation bay of the participating hospital. This programme was designed to be cost‐effective and easily replicable in resource‐limited settings.

The TTRRT programme has three phases. The first phase is a web‐based pre‐programme survey, the second is participant attendance and assessment and the third phase is a web‐based post‐programme evaluation survey.

### Design and implementation

**Figure 1:**
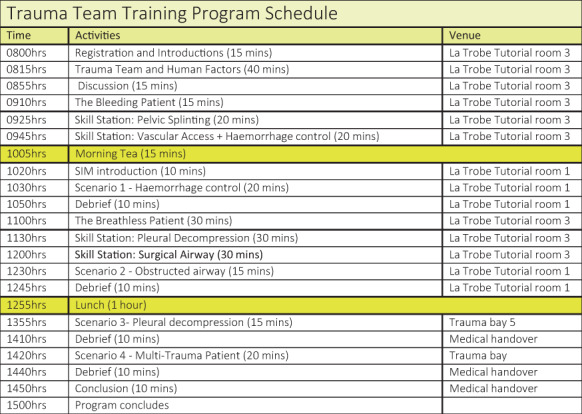
Programme schedule example.

Participants were directed to prescribed ‘pre‐learning’ on the Trauma Victoria website (http://trauma.reach.vic.gov.au/) prior to face‐to‐face training to improve core knowledge. Face‐to‐face training occurred over 7 h and involved didactic lectures, skills station and low‐fidelity simulation (Fig. 1). Each face‐to‐face session was facilitated by two trauma critical care and/or trauma surgical consultants and two critical care trained emergency nurses. During the session, participants were divided into groups consisting of anaesthesia, trauma/surgical medical, emergency medical and emergency nursing with varying years of experience.

### Session content

**Pre‐learning**: Prior to the programme, participants received an email containing details of the session. Participants were directed to the Trauma Victoria website. The website contains the standardised guidelines for trauma patient management within the VSTS. Participants were also provided with a manual that covered the roles and responsibility of each speciality of clinician in a trauma team. This was designed to provide all participants with same level of understanding and knowledge of team performance prior to the training session.

**Figure 2:**
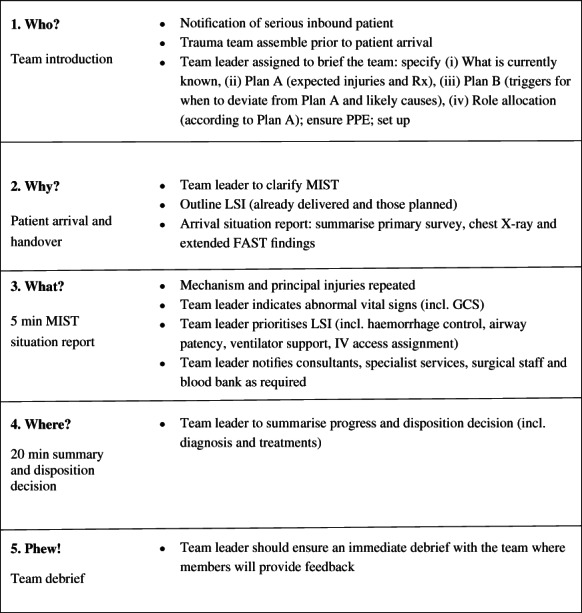
Trauma Team Time‐out.^17^

**Lectures**: Sessions started with a lecture covering trauma team dynamics and human factors,^16^ including the introduction of Trauma Team Time‐out Model (Fig. 2).^17^ This was followed by a facilitated group discussion around participant experiences and perceived challenges to high‐quality team‐based trauma reception and resuscitation.

Subsequent sessions covered ‘the bleeding patient’, focussing on early team‐based haemorrhage control followed by ‘the breathless patient’ focusing on airway and breathing management. Both lectures addressed the common ‘pearls and pitfalls’ of team‐based care within these topic areas.

**Skill stations:** Four skill stations were set up for the programme day: haemorrhage control and vascular access, tourniquet and pelvic binder application, pleural decompression and surgical airway. The sessions were based on the set of procedures considered to be essential life‐saving interventions in the setting of major trauma.^18^ Debriefing was included in the skill stations.

**Simulated scenarios**: The programme used low‐fidelity simulation that would be easily replicated in low‐resource environment. Local volunteer nursing and medical students were recruited for each session. The student was given specific instruction to act out each scenario with moulage make up applied to simulate real‐life injuries.

Theoretical knowledge for each scenario was covered in the lecture and skill station prior to the simulated scenarios, allowing for repetition of learning in the simulated work environment. Participants were instructed to provide leadership and care as they would in a clinical situation and were encouraged to use the Trauma Team Time‐out Checklist^9^, ^17^ including debriefing at the end of the scenario. Each simulation has six participants – three doctors and three nurses. The rest of the participants observed the simulation and provided feedback to the participating group at the end of the simulation.

**Observation and debrief**: Structured debriefing occurred after each simulated scenario. Goals of the debrief were to explore key aspects of teamwork, communication, decision making, leadership and followership. Observers and participants were encouraged to participate in the discussion. Each debriefing duration was adjusted according to the content discussed, with an aim of 20 min for each scenario.

### Measurement of outcomes

Each participant received two surveys: one pre‐session and one post‐session (2 weeks prior to the programme, and within 2 weeks after completion of the programme). Both surveys contained the same themed questions, worded with appropriate tense. These surveys aimed to evaluate participant confidence in trauma team leadership, clinical decision‐making and in delivering lifesaving interventions.

The primary outcome was a binary variable of enabling of leadership within a trauma team defined as the proportion of participants who were not participating in leadership roles prior to the programme and who subsequently undertook leadership roles after the programme. Secondary outcome variables included participant perceived confidence in trauma team leadership, confidence in raising issues of clinical risk and confidence in management of critical injuries, including procedural skill performance.

Ethics approval for collection of participant experience and surveys was granted by Monash University, Melbourne, Australia. Participants were provided with information regarding the study design and consented to take part before undertaking the pilot course. Participants who declined to take part in the study were still allowed to participate in the course.

Survey questions focussed on prior experience in acute trauma care, prior experience in trauma team training, confidence in both trauma team leadership and participation (followership) and confidence in decision making around and performance of lifesaving interventions in acute trauma care. Participant discipline (e.g., nursing, medical) and years of experience post‐graduation were also collated.

**Analysis**: Participant characteristics were summarised using count and proportions. The primary outcomes for paired responses pre‐ and post‐programme were assessed using differences across the population as well as within subjects. Associations of the primary outcome with prior experience were assessed using univariable logistic regression analysis and presented using odds ratios and 95% confidence intervals. Results from the ordinal numeric scale of confidence were summarised using medians and compared using Wilcoxon rank‐sum test. A *P*‐value of <0.05 was defined to be statistically significant. All analyses were conducted using Stata v 15.1 (College Station, TX, USA).

## Results

There were 252 participants, and 120 (47.6%) responses were received. The 120 responders' characteristics were representative of those of the total participants. Baseline characteristics of the responding participants are listed in Table 1.

**TABLE 1:** Baseline characteristics

Characteristic	*n* (%)
Setting of work	
Major trauma centre	61 (50.8%)
Regional trauma centre	26 (21.7%)
Rural ED	30 (25.0%)
Other	3 (2.5%)
Role in trauma team	
Critical care nurse	34 (28.3%)
Registered nurse	26 (21.7%)
EM doctor	18 (15.0%)
Other specialist doctor	23 (19.2%)
Doctor‐in‐training	19 (15.8%)
Years of clinical practice	
0–5	30 (25.0%)
6–10	45 (37.5%)
11–15	18 (15.0%)
>15	27 (22.5%)
Used Trauma Vic guidelines	66 (55.0%)
Formal trauma training	46 (38.3%)
Trauma cases managed in previous 4 weeks	
0–10	102 (85.0%)
11–20	10 (8.3%)
>20	8 (6.7%)

The survey demonstrated improvements in enabling leadership of trauma teams with a significant improvement of proportion of participants who led trauma teams after participation in the programme from 53 (44.2%) to 74 (61.7%; *P* = 0.007). There were 29 (24.4%; 95% CI 17.0–33.0) participants who achieved the primary outcome of undertaking team leading roles after participation in the programme, after not being leaders prior to the programme. No differences were observed among 83 (69.2%) participants while eight (6.7%) participants reverted to not being team leaders after the programme. There was no association of the primary outcome with the type of participant role (nurse or doctor), years of experience and trauma experience in the 4 weeks prior to the programme (Table 2).

**TABLE 2:** Association of new leadership roles with participants and experience

Variable	OR (95% CI)	*P*‐value
Role		
Nurse	Ref	
Doctor	0.44 (0.18–1.05)	0.06
Years of experience		
<5 years	Ref	
5–10 years	1.7 (0.61–4.6)	0.32
>10–15 years	0.34 (0.06–1.8)	0.21
>15 years	0.23 (0.04–1.20)	0.08
Trauma cases in previous 4 weeks		
0–10	Ref	
>10–20	2.26 (0.59–8.70)	0.24
>20	1.13 (0.21–5.99)	0.88

**Figure 3:**
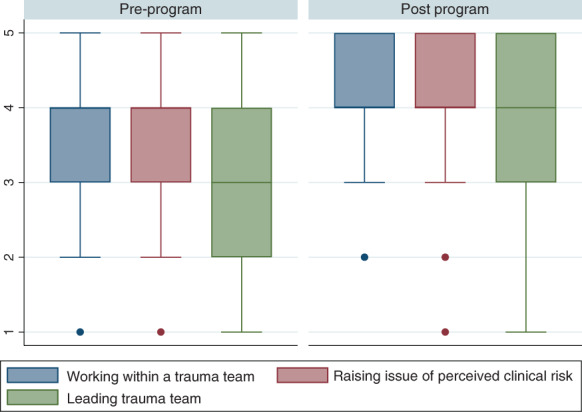
Improvements in key outcome variables after participation in the Alfred Trauma Team Reception and Resuscitation Training programme. 1: least confident; 5: most confident.

Among responders to the question, there were 75 (63.6%) participants who reported increased confidence in working within a trauma team after the programme, with 39 (33.1%) reporting no change and four (3.4%) participants reporting reduced confidence. There were 72 (60.5%) participants who reported increased confidence in being able to raise a concern of perceived clinical risk, 42 (35.3%) reported no change, while five (4.2%) reported reduced confidence. With regard to leading a trauma team, 83 (70.3%) reported increased confidence after the programme, 30 (25.2%) reported no change whereas five (4.2%) reported reduced confidence (Fig. 3). Significantly improved confidence was demonstrated in all key topics (Table 3).

**TABLE 3:** Primary and secondary outcomes

	Pre‐programme (*n* = 120)	Post‐programme (*n* = 120)	*P*‐value
Leading a trauma team	53 (44.2%)	74 (61.7%)	0.007
Perceived confidence levels			
Working withing a team	4 (3–4)	4 (4–5)	<0.001
Raising perceived clinical risk	4 (3–5)	4 (4–5)	<0.001
Leading a trauma team	3 (2–4)	4 (3–5)	<0.001
Managing shock	4 (3–4)	4 (4–5)	<0.001
Managing severe airway injury	3 (2–4)	4 (4–5)	<0.001
Managing severe traumatic brain injury	3.5 (3–4)	4 (4–5)	<0.001
Managing chest trauma	3 (2.5–4)	4 (4–5)	<0.001
Managing abdominal trauma	3 (3–4)	4 (4–5)	<0.001
Managing severe limb trauma	3 (3–4)	4 (4–5)	<0.001
Managing severe pelvic trauma	3 (2–4)	4 (4–5)	<0.001
Managing severe burns	3 (2–4)	4 (3–4)	<0.001
Managing severe spinal injury	3 (2–4)	4 (4–5)	<0.001

## Discussion

Despite the reported benefits of trauma teams, a standardised approach to team‐based trauma reception and resuscitation has yet to be agreed. Advanced Trauma Life Support (ATLS©) version 10 continues to focus on a serial approach to trauma care suited to the single provider^19^ and the World Health Organization trauma checklist^20^ provides a ‘safety’ framework. However, both do not define the workflow and activities of a high‐functioning trauma team. Work by Fitzgerald *et al*.^17^ attempts to define these activities in greater depth. However, further research is required to understand its applicability in hospitals with different levels of resources and the impact on patient outcomes.

This programme was successful in enabling clinicians to lead trauma teams in a standardised fashion in a simulation environment. Leadership was developed independent of the participant's role, years of experience in their specialty or experience in trauma resuscitation. Perceived confidence in leadership and management of key trauma topics demonstrated significant improvement. These findings indicate the adoption of clinically transferrable non‐technical skills in the practice of trauma resuscitation after TTRRT and therefore, supports wider programme dissemination.

Pre‐programme material aimed to empower all participants with the same level of knowledge. Didactic lectures and skills stations were designed to correlate with briefing prior to the simulated stations. Previous studies had demonstrated that by providing participants with effective orientation and creating a psychologically safe environment, the participants' fear and anxiety was lowered and enhanced the learning experience.^21^

The immediate perception of improved confidence observed in the present study is consistent with reports from other simulation programmes. For optimal benefits, it has been recommended that simulation must be integrated into a comprehensive curriculum and not considered as a stand‐alone system.^22^ Performance and confidence in procedures are known to decrease at 3–6 months after training, with total loss expected at 2–4 years after training, irrespective of experience.^23^ Increased emphasis towards maintenance and booster training is therefore indicated, along with surveillance of confidence and practice over the longer term. Although the optimal rate of refresher courses requires further research, 2‐year refresher programmes would be a reasonable starting point.

Although there were uniform improvements in participants' ability to deliver improved care, the translation of such programmes to improved patient benefits remains unknown. The effect of trauma team training has yet to be associated with mortality benefits, but improvements in processes such as time to theatre and imaging have been observed.^24^

A decrease in confidence to lead trauma teams was observed in a small proportion of participants. This is consistent with a previous study that demonstrated an initial reduction in provider confidence, possibly due to a relative lack of experience in the high‐acuity trauma scenarios. In effect, new knowledge gained through training may unmask previously ‘unconscious inadequacy’ among participants.^25^ The skill stations and simulated scenarios were a new experience for most participants – making them aware of the challenges including difficulty in clear communication and cohesive teamwork in this environment. Another source of the initial decrease in provider confidence may have been the post‐simulation debriefing.^26^ The advocacy‐inquiry technique used for debriefing probes into the origins of errors by clearly identifying errors to the team and encouraging discussion from multiple team‐member perspectives. Although the focus was not on individual errors, debriefing as a team and open discussion of errors may have increased anxiety and decreased confidence.

Only a small proportion of providers within the state trauma system were captured by this initial programme, limiting generalisability. Also, only half of the participants completed the post‐programme feedback. The programme, however, was designed for ready translation to all trauma receiving hospitals including receiving hospitals in developing countries. The self‐reported, perceived improvement in confidence to lead teams and perform procedures cannot yet be extrapolated to actual performance.

## Conclusion

It was possible to initiate a standardised, multi‐disciplinary trauma team training programme in a large, mature trauma system. Short‐term positive changes in learner‐perceived confidence in team leadership, decision‐making and procedural skills were observed. These findings demonstrate the potential of such programmes to substantially improve the standardisation and practice of team‐based trauma reception and resuscitation and support wider dissemination. Longitudinal research is indicated to examine the association between trauma team training programmes and staff and patient outcomes.

## Data Availability

The data that support the findings of this study are available from the corresponding author upon reasonable request.
